# PACS-2 functions in colorectal cancer

**DOI:** 10.18632/aging.101489

**Published:** 2018-06-22

**Authors:** Marie Kveiborg, Gary Thomas

**Affiliations:** 1 Biotech Research and Innovation Centre (BRIC), University of Copenhagen, Copenhagen, Denmark;; 2 Department of Microbiology and Molecular Genetics, University of Pittsburgh School of Medicine, Pittsburgh, PA 15213, USA

**Keywords:** PACS-2, colorectal cancer, EGFR, ADAM17, apoptosis

Colorectal cancer (CRC) development is a step-wise process initiated by the transformation of epithelial cells in the intestinal crypts, followed by the accumulation of multiple genetic events driving adenoma to carcinoma progression. In this way, genomic instability underlies the majority of CRCs and chromosomal alterations such as translocations, amplifications and deletions are frequently seen.

In human, the gene encoding the intracellular sorting protein phosphofurin acidic cluster sorting protein-2 (PACS-2) is located near the telomere at 14q32.33, a locus prone to allelic loss in sporadic CRC. Accordingly, PACS-2 protein expression was found to be lost or greatly reduced in tumor tissue from approximately half the CRC patients analyzed [1]. Searching the Cancer Genome Atlas (http://cancergenome.nih.gov), *Pacs2* is found mutated in 5% of CRC cases, with the two most frequent somatic mutations leading to inframe deletions. Also, *Pacs2* was identified as one of 25 genes that can be used to distinguish different CRC stages [2].

With the described correlations, the question arising is whether *Pacs2* loss is a driver or passenger mutation in the development of CRC? To answer this question, depletion of *pacs2* in mice is a useful experimental model. PACS-2-deficient mice do not develop spontaneous neoplastic lesions in the intestine, and we recently reported that inactivation of one allele of the APC gene (ApcMin) – a commonly used model of human colon cancer – in PACS-2-deficient mice had no apparent consequence for adenoma development [3].

Rather, mice lacking PACS-2 expression exhibit reduced activity of the epidermal growth factor receptor (EGFR) and lower proliferative index in the intestinal epithelium [4]. Likely explaining this observation, we previously showed that PACS-2 directs recycling of a disintegrin and metalloprotease (ADAM)17 towards the cell surface to sustain shedding of EGFR ligands and consequent EGFR activation [4]. Ligand-induced EGFR signaling stimulates cell proliferation, differentiation, and survival and it plays an important, yet complex role in the development and progression of CRC – e.g. deletion of EGFR from myeloid cells, but not intestinal epithelial cells protects mice from inflammation-induced intestinal cancer and ApcMin-dependent intestinal tumorigenesis [5]. ADAM17 is required for EGFR-induced intestinal tumors, apparently by shedding EGFR ligands to activate EGFR-dependent interleukin (IL)-6 synthesis in colonic myloid cells, which via IL-6 trans-signaling induces epithelial tumor formation [6].

The explanation why PACS-2 loss has no apparent effect on intestinal tumor formation may lie in the complex roles of PACS-2 as a phosphorylation-state dependent molecular switch that mediates either anti-apoptotic or pro-apoptotic signaling [7]. In response to DNA damage, Akt-phosphorylated PACS-2 promotes pro-survival signaling; it binds the DNA damage kinase ATM to promote the NF-kB-dependent induction of anti-apoptotic Bcl-xL and it inhibits the class III deacetylase SIRT1 to promote the p53-dependent induction of cyclin dependent kinase inhibitor p21 (CDKN1A). By contrast, the death ligand TRAIL triggers dephosphorylation of PACS-2, which switches the multi-functional protein to become a pro-apoptotic effector. Dephosphorylated PACS-2 shuttles the BH3-only proteins Bid and Bim to mitochondria and lysosomes, respectively. These steps trigger organellar membrane permeabilization necessary to activate executioner caspases and induce cell death. Thus, analysis of Pacs2 loss may not adequately determine whether it is a driver or passenger in CRC. Such a conclusion awaits gene replacement studies that separate the complex anti- and pro-apoptotic roles for PACS-2 in maintaining cell homeostasis.
